# Occurrence of Anti-Drug Antibodies against Interferon-Beta and Natalizumab in Multiple Sclerosis: A Collaborative Cohort Analysis

**DOI:** 10.1371/journal.pone.0162752

**Published:** 2016-11-02

**Authors:** Delphine Bachelet, Signe Hässler, Cyprien Mbogning, Jenny Link, Malin Ryner, Ryan Ramanujam, Michael Auer, Poul Erik Hyldgaard Jensen, Nils Koch-Henriksen, Clemens Warnke, Kathleen Ingenhoven, Dorothea Buck, Verena Grummel, Andy Lawton, Naoimh Donnellan, Agnès Hincelin-Mery, Dan Sikkema, Marc Pallardy, Bernd Kieseier, Bernard Hemmer, Hans Peter Hartung, Per Soelberg Sorensen, Florian Deisenhammer, Pierre Dönnes, Julie Davidson, Anna Fogdell-Hahn, Philippe Broët, on behalf of the ABIRISK Consortium

**Affiliations:** 1 CESP, Université Pa ris-Sud, UVSQ, INSERM, Université Paris-Saclay, Villejuif, France; 2 Karolinska Institutet, Department of Clinical Neuroscience, Clinical Neuroimmunology, Stockholm, Sweden; 3 KTH—Royal Institute of Technology, Stockholm, Sweden; 4 Department of Neurology, Innsbruck Medical University, Innsbruck, Austria; 5 Danish MS Center, Department of Neurology, Copenhagen University Hospital Rigshospitalet, Copenhagen, Denmark; 6 Danish Multiple Sclerosis Registry, Copenhagen University Hospital, Rigshospitalet, Copenhagen, Denmark; 7 Department of Clinical Epidemiology, University of Aarhus, Aarhus, Denmark; 8 University of Düsseldorf, Medical Faculty, Department of Neurology, Düsseldorf, Germany; 9 Department of Neurology, Technische Universität München, Munich, Germany; 10 GlaxoSmithKline, Uxbridge, Middlesex, United Kingdom; 11 IPSEN, Slough, Berkshire, United Kingdom; 12 Sanofi, Chilly-Mazarin, France; 13 INSERM UMR 996, Univ. Paris-Sud, Faculty of Pharmacy, Université Paris-Saclay, Châtenay-Malabry, France; 14 Munich Cluster for Systems Neurology (SyNergy), Munich, Germany; 15 SciCross AB, Skövde, Sweden; 16 Assistance Publique—Hôpitaux de Paris, Hôpital Paul Brousse, Villejuif, France; University of Oxford, UNITED KINGDOM

## Abstract

Immunogenicity of biopharmaceutical products in multiple sclerosis is a frequent side effect which has a multifactorial etiology. Here we study associations between anti-drug antibody (ADA) occurrence and demographic and clinical factors. Retrospective data from routine ADA test laboratories in Sweden, Denmark, Austria and Germany (Dusseldorf group) and from one research study in Germany (Munich group) were gathered to build a collaborative multi-cohort dataset within the framework of the ABIRISK project. A subset of 5638 interferon-beta (IFNβ)-treated and 3440 natalizumab-treated patients having data on at least the first two years of treatment were eligible for interval-censored time-to-event analysis. In multivariate Cox regression, IFNβ-1a subcutaneous and IFNβ-1b subcutaneous treated patients were at higher risk of ADA occurrence compared to IFNβ-1a intramuscular-treated patients (pooled HR = 6.4, 95% CI 4.9–8.4 and pooled HR = 8.7, 95% CI 6.6–11.4 respectively). Patients older than 50 years at start of IFNβ therapy developed ADA more frequently than adult patients younger than 30 (pooled HR = 1.8, 95% CI 1.4–2.3). Men developed ADA more frequently than women (pooled HR = 1.3, 95% CI 1.1–1.6). Interestingly we observed that in Sweden and Germany, patients who started IFNβ in April were at higher risk of developing ADA (HR = 1.6, 95% CI 1.1–2.4 and HR = 2.4, 95% CI 1.5–3.9 respectively). This result is not confirmed in the other cohorts and warrants further investigations. Concerning natalizumab, patients older than 45 years had a higher ADA rate (pooled HR = 1.4, 95% CI 1.0–1.8) and women developed ADA more frequently than men (pooled HR = 1.4, 95% CI 1.0–2.0). We confirmed previously reported differences in immunogenicity of the different types of IFNβ. Differences in ADA occurrence by sex and age are reported here for the first time. These findings should be further investigated taking into account other exposures and biomarkers.

## Introduction

The introduction of biotechnology-derived proteins (BPs) has been a critical step forward in the treatment of multiple sclerosis (MS), with BPs such as interferon beta (IFNβ) registered since the 1990s and natalizumab available since 2006 in the EU. Natalizumab is mainly used as a second line treatment for patients with relapsing-remitting MS (RRMS) that do not respond to treatment with first line drugs such as IFNβ. Failure of response to therapy may occur due to an unwanted immune response against the BP. This immunological reaction to BPs is not fully understood and the fraction of patients developing anti-drug antibodies (ADA) has varied from 2 to 47%, depending upon either the product or the laboratory methods and cut-off used to define antibody positivity [1].

The consequences of this immunological reaction range from transient ADA to high titer ADA with clear clinical effects [2,3]. In particular, the occurrence of ADA may decrease the efficacy of BPs by neutralizing their activity or modifying their drug clearance and ADA may also be associated with BP-specific hypersensitivity reactions [4]. Clinically relevant neutralizing ADA (NAbs) against IFNβ usually develop between 9 and 18 months after start of therapy [5]. Patients who have been persistently NAb negative during the first 2 years of IFNβ therapy only rarely become NAb positive [6]. For natalizumab, the antibodies are detected in approximatively 9% of treated patients with 3% that are transient [7]. These ADA develop quickly, within 4 months following the first infusion of treatment [8]. In establishing the used ADA assay, the cut-point set for ADA positivity by enzyme-linked immunosorbent assay (ELISA) correspond to neutralizing antibody positive by flow cytometry blocking assay [7].

Many factors (patient-, disease- or product-related) may influence the risk of BP immunogenicity but the relative contribution of these factors to the development of ADA is not currently understood. The ability to identify patients at high risk for ADA and subsequent lack of treatment efficacy or hypersensitivity reactions would allow better personalized therapy. The immunogenic potential of BPs can only be definitively assessed in human studies. Previous investigations have identified oligoclonal band negativity as a protective factor [9], specific HLA haplotypes as risk factors for IFNβ ADA [10–13], and smoking as a risk factor for both IFNβ and natalizumab ADA [7–9].

In order to carry out an unbiased evaluation of the relationships between bio-clinical factors and the occurrence of ADAs, we selected a target population that was extracted from the whole ABIRISK tranSMART database based on five European MS cohorts and that is composed by BP naive adult patients with biological samples being taken within the first two years of treatment.

The present study gathers data from four European central laboratories and one research center routinely performing ADA tests in order to identify patient-related and treatment-related factors associated with ADA development in IFNβ- and natalizumab-treated MS patients using a time-to-event analysis.

## Patients and Methods

### Patients

The present study is a collaborative analysis of historical data collected from a number of European MS cohorts under the leadership of the ABIRISK EU-consortium, standardized into a common format and grouped into the ABIRISK tranSMART database including data from national routine ADA test laboratories in Sweden, Denmark, Austria and Germany (Dusseldorf group) and from one research study in Germany (Munich group).

In Sweden, Germany and Denmark an ethical permission for the analysis of anonymized patient retrospective data was required. For the Swedish cohort the study was approved by the Regional Ethics Committee in Stockholm, Sweden (http://www.epn.se/en/start/the-organisation/), and approval for export of data from the national Multiple Sclerosis Registry (MSreg) was given by the Research Board of the Swedish MS Society. For the German cohorts, the study was approved by the Ethics Committee of the School of Medicine of the Technical University of Munich and the Ethics Committee of the medical faculty of the University of Dusseldorf. The Danish MS-cohort collected in the Danish Multiple Sclerosis Biobank was approved by the Regional Scientific Ethics Committee in Copenhagen and Frederiksberg, Denmark, and approval for use of data was given by the Danish Data Protection Agency in the Capital Region, Denmark. In Austria the Ethics Committee judged that for retrospective analyses based on anonymized data that were collected during routine procedures, an ethical approval was not required.

In total the database contained 20,465 patients from the four countries and ADA data of 42,843 samples together with treatment history, type of ADA assay, age and sex. Data about anti-natalizumab ADA tests were available only in reference laboratories from Sweden, Austria and Denmark.

Populations eligible for inclusion in the IFNβ and natalizumab historical longitudinal analyses were selected based on the following criteria: (i) for IFNβ, naive to IFNβ prior to the IFNβ for which the first NAb test is available, (ii) for IFNβ and natalizumab, date of first BP dose administration known (at least with a month precision), (iii) for IFNβ, at least one NAb test result available after first dose and before 24 months of treatment, (iv) for natalizumab, at least one ADA test result available after baseline and before 12 months of treatment, and (v) for IFNβ and natalizumab, age at date of first BP dose more than 18 years.

Due to the low sample size obtained from the Swiss and Spanish cohorts, we focused on the cohorts from Sweden, Austria, Denmark, and Germany (Dusseldorf and Munich groups). IFNβ-treated MS patients tested for ADA from 2002 to 2013 in Sweden, from 1996 to 2014 in Austria, from 2009 to 2014 in Denmark, from 2008 to 2014 in the German reference laboratory (Dusseldorf group) and from 2005 to 2008 in the German research study (Munich group) were included in the time-to-event analysis. Eligible natalizumab-treated patients were tested from 2006 to 2013 in Sweden, from 2007 to 2014 in Austria and from 2007 to 2014 in Denmark.

### The ABIRISK tranSMART database

The ABIRISK database integrates immunogenicity data from a number of European countries in a well-structured format. The data is harmonized over different cohorts and the format is based on the CDISC standards (http://www.cdisc.org/). Where no previous variable description can be found in CDISC, a local variable description is used. Data was prepared by data custodians using a data load plan describing the variable semantics and format. Anonymized data was uploaded into the ABIRISK database, which is based on the tranSMART platform [14]. The tranSMART platform is an open-source knowledge management platform for translational science, supported by a large number of organizations (http://www.transmartfoundation.org/). tranSMART provides tools to explore data, select specific cohorts and do basic statistics. It is also possible to export data for further analysis in tools like SAS and R.

### Biological samples testing

The reference laboratories received serum samples for analysis of neutralizing antibodies.

According to the countries, ADA screening was either mandatory for all BP-treated MS patients as in Denmark since 1996, sponsored by the pharmaceutical companies and so widely performed as in Sweden from 2003 to 2006 (thereafter performed ad hoc at the request of the neurologists) or always performed ad hoc at the request of the neurologists as in Austria and Germany (Dusseldorf). In the Munich study, ADA testing was performed in one single instance for research purposes during an outpatient visit in the period 2003 to 2008.

In each laboratory, different methods to analyze ADA against IFNβ were used. Some laboratories search for both binding ADA and NAbs (Austria, Germany, Denmark) while others only tested for NAbs (Sweden). Since these latter are more frequently persistent and clinically relevant than binding ADA, in the present study only ADA determined to be NAbs against IFNβ were considered.

In Sweden, three different NAb assays were performed across time: patient samples sent to the laboratory in 2003–2006 were analyzed with the myxovirus resistance protein A (MxA) assay (MPA) [15] while later samples were analyzed with the MxA gene expression assay (MGA) [16,17]. Since 2012, samples were analyzed with the commercially available *iLite* anti-Human IFNβ-1a bioassay (Biomonitor/Eurodiagnostica). In a recent publication a strong correlation of NAb titers was found on a series of 118 serum samples titrated with the MPA and the MGA assay in the Swedish central laboratory [12]. There were also significant correlations between NAb titer levels measured with MGA and *iLite* [18]. For the three assays, titers were adjusted using the Kawade formula [19]. NAb-positive screening samples were further titrated and NAb titers were expressed as 10-fold reduction units per milliliter (TRU/ml) and the patients were classified according to the following categories: negative (<10 TRU/ml), low positive (≥ 10–50 TRU/ml), medium positive (>50–200 TRU/ml), and high positive (>200 TRU/ml).

In Austria, three different NAb assays were also performed across time: the MPA and the MGA assays, as well as the Luciferase assay (LUC) where NAb were measured using human fibrosarcoma cells that express IFN-β receptors on their surface and were stably transfected with a luciferase reporter gene cassette [20,21]. The cut-off for positivity for all NAb-tests (MPA, MGA and LUC) was 20 TRU/ml, whereby 20–99 TRU/ml were considered low positive and titers beginning from 100 TRU/ml high positive.

In Denmark, only results from the LUC method were applied. NAb-positivity was defined by a titer ≥ 20 TRU/ml.

In Dusseldorf, ADA were first screened in the ELISA. Then NAbs were usually only tested with LUC in case of a positive ADA testing. The patients were classified according to the following categories: negative (<20 TRU/ml), low positive (≥ 20–100 TRU/ml) and high positive (>100 TRU/ml).

In Munich, ADA were first tested using a capture ELISA as described by Pachner et al. [22]. Next antibodies were defined as NAb if MxA induction was decreased by more than 50% compared with newly treated antibody-negative control donors. A cross-validation study on 247 samples was performed between Austria and Munich and has shown an agreement of 96% for classifying samples in NAb positive (>30 TRU/ml) versus NAb negative (personal communication from F. Deisenhammer).

In our analyses, to define clinically relevant NAb-positivity against IFNβ, we kept the cut-off used in each reference laboratory (≥200 TRU/ml in Sweden, ≥100 TRU/ml in Austria, ≥20 TRU/ml in Denmark, ≥100 TRU/ml in Germany–Dusseldorf, and >50% inhibition in Munich).

Concerning natalizumab, the same assay method undergoing regular proficiency testing was used in Sweden, Austria and Denmark. Antibodies were measured using a bridging ELISA method as described in the Biogen Idec protocol [7].

For ease of reading, we use "ADA" as a generic term in the remainder of the text for either NAbs concerning IFNβ-treated patients or ELISA-detected ADA concerning natalizumab-treated patients.

### ADA outcome

In this work, we focused on a two-year ADA occurrence window after the start of therapy. To take into account that ADA occurrence is a dynamic event which appears mainly within this two-year window but can be censored, we performed time-to-event analyses. The outcome was the time between the date of the first BP dose and the first time of ADA occurrence (positive ADA). Patients without ADA occurrence were censored at the date of their last clinic visit, the end of treatment or at the last visit before a drug switch.

In practice, the ADA occurrence process was not under continuous monitoring. As a consequence, a positive ADA result indicated that ADA have been produced during a particular time interval but the exact event time of the ADA occurrence was unknown. We only knew that it occurred between two time points: the time of the last negative laboratory test and the time of the first positive laboratory test. For the analyses, we considered interval-censored survival methods that explicitly took into account the information obtained for each patient.

### Statistical analysis

For both therapies IFNβ and natalizumab, we present results of separate analyses in each cohort and pooled analysis.

The non-parametric estimator for the cumulative probability distribution function under interval censoring was computed as the complement of the survival estimator obtained using the self-consistency or Turnbull’s algorithm [23]. The cumulative probability distribution function curves were plotted for each BP (IFNβ and natalizumab separately) and according to categorical variables (type of therapy, age group, gender, month of first treatment and month of birth). In order to compare these curves, p-values for rank-based tests adapted for interval censoring [23–25] are presented.

For the univariate and multivariate analyses, we considered the Cox proportional hazards model via a multiple imputation strategy for the unobserved survival times as proposed by Pan [26].

Univariate Cox model analyses were carried out to assess the influence of clinical variables on the time of occurrence of ADA. Hazard ratios (HR) and 95% confidence intervals (CIs) were determined and reported. We investigated the impact of age, sex, month of therapy start and month of birth. Previous IFNβ therapy before natalizumab were also explored (last IFNβ taken, first IFNβ taken, last ADA status during IFNβ therapy, total duration of IFNβ therapy and number of different IFNβ therapies).

All variables associated with the time to ADA occurrence in univariate analysis (with p-value less than 0.20) were considered for the multivariate analysis using the Cox proportional hazards model. Hazard ratios (HR) and 95% confidence intervals (CIs) were determined and reported.

Analyses were performed using R software (version 3.0.2 software) with "interval", "Icens" and "MIICD" packages.

## Results

**Fig 1:**
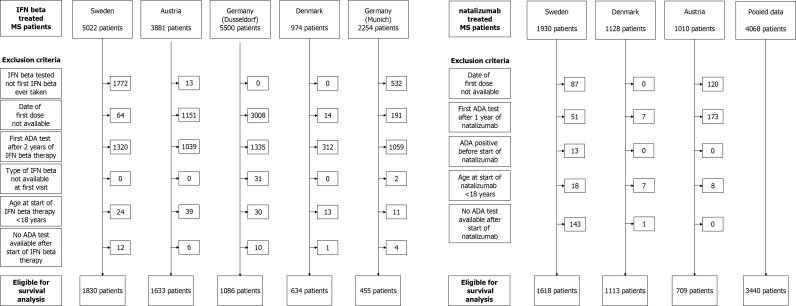
Inclusion flow chart. Flow chart of patients by country for IFNβ (left side) and natalizumab (right side) treated patients.

The flowchart (Fig 1) gives the details of the patients eligible in each cohort according to the chosen inclusion criteria for IFNβ and natalizumab.

Among the 17,631 available IFNβ-treated patients in the database, 5,638 eligible patients (32%) were considered for the survival analysis. For natalizumab, the pooled eligible cohort consisted of 3,440 patients (85% of the patients available in the database).

We compared included patients treated with IFNβ to those not included with respect to age at therapy start, sex, type of first IFNβ and ADA status for the first test performed. Regarding the BPs, the fraction of included patients was higher for IFNβ-1a intramuscular (i.m.) as compared to the other type of treatment (S5 Table). There was also a significant difference in age at start of therapy in Germany-Dusseldorf (fewer patients older than 40 were included) and ADA status at first visit differed between included and excluded in Sweden, Austria and Germany-Dusseldorf (S5 Table). Concerning natalizumab-treated patients, a lower proportion was excluded. They did not differ from included patients, except for ADA status at first test available in Sweden: more excluded patients were ADA positive. In most of the cases, this first test is a pre-treatment sample. All the patients with baseline ADA positive tests were excluded from analysis.

In this selected historical cohort, the median number of tests for ADA against IFNβ done for one patient was 2 (max = 7) in Sweden, 2 (max = 41) in Austria, 2 (max = 7) in Denmark, 1 (max = 7) in Germany—Dusseldorf group, and 1 in Germany–Munich group (current status data). Concerning natalizumab, the median number of ADA tests done for one patient was 4 (max = 10) in Sweden, 2 (max = 50) in Austria, and 3 (max = 9) in Denmark.

In the reference laboratories, the first IFNβ ADA test was performed around one year after first dose of therapy in Sweden, Austria and Germany, and 6 months after first dose of therapy in Denmark. Thereafter tests were done approximatively every 6 months in each country.

Concerning natalizumab, time interval between two visits was approximatively from 3 to 6 months.

### Univariate analyses

#### Results for IFNβ

**Fig 2:**
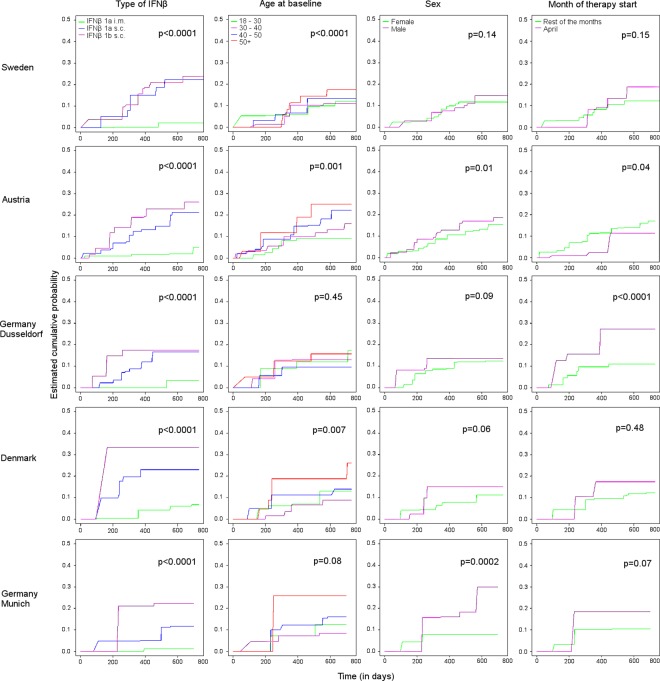
Cumulative probability of developing anti-IFNβ ADA. Estimated cumulative probability of developing anti-IFNβ ADA by cohort and by categorical variables with p-values for rank-based tests adapted for interval censoring.

According to the univariate analyses, the type of IFNβ treatment was strongly associated with the rate of ADA occurrence (p < 0.0001 in each cohort) (Fig 2).

As expected most of the ADA occurred within two years of IFNβ therapy. Of the two different routes of administration for IFNβ-1a, the one with a single weekly intramuscular (i.m.) dose was less immunogenic with 1.1 to 4.2% (95% confidence intervals (CI) ranging from 0 to 6.8%) of the patients who developed ADA before 18 months of therapy as compared to 11.1 to 23.0% (95% CI ranging from 1.0 to 30.7%) for the patients with three weekly subcutaneous (s.c.) doses (Table 1). Patients treated with IFNβ-1b s.c. every other day had an even higher rate of ADA development before 18 months of therapy: 17.4 to 33.3% (95% CI ranging from 0 to 51.7%).

**Table 1:** Estimated cumulative probabilities with 95% confidence interval of ADA development before 18 months of IFNβ therapy.

		Sweden		Austria		Germany (Dusseldorf)		Denmark		Germany (Munich)	
		(cut-off = 200 TRU/mL)		(cut-off = 100 TRU/mL)		(cut-off = 100 TRU/mL)		(cut-off = 20 TRU/mL)			
		(N = 1830)		(N = 1633)		(N = 1086)		(N = 634)		(N = 455)	
		n	% 95% CI	n	% 95% CI	n	% 95% CI	n	% 95% CI	n	% 95% CI
Type of	IFNβ-1a i.m.	877	2.1 [0.0–3.4]	607	2.0 [0.7–5.3]	308	3.3 [0.0–6.4]	438	4.2 [0.0–6.8]	130	1.1 [0.0–3.6]
IFNβ	IFNβ-1a s.c.	461	22.3 [13.4–25.5]	592	15.0 [9.7–19.9]	380	16.7 [8.2–20.7]	165	23.0 [14.5–30.7]	154	11.1 [1.0–16.9]
	IFNβ-1b s.c.	492	21.1 [15.0–26.2]	434	22.8 [16.4–27.0]	398	17.4 [10.0–20.6]	31	33.3 [0.0–51.7]	171	22.4 [14.5–32.0]
Age at	18–30	389	9.9 [5.4–14.7]	491	9.3 [6.0–11.7]	373	11.9 [5.7–15.3]	150	13.0 [5.0–19.5]	30	12.5 [4.3–22.5]
baseline	30–40	551	10.3 [3.2–13.2]	574	11.8 [7.8–15.4]	339	13.1 [6.8–16.9]	219	6.6 [2.7–11.7]	146	8.5 [2.7–16.4]
	40–50	564	13.5 [4.4–16.7]	416	18.2 [12.4–25.4]	263	9.6 [3.6–13.4]	179	11.2 [7.9–19.2]	150	12.2 [0.0–17.8]
	50+	326	14.4 [8.3–19.1]	152	25.0 [12.2–33.0]	111	15.8 [5.9–26.7]	83	18.8 [12.3–37.4]	129	25.9 [0.0–40.7]
Sex	Female	1292	11.7 [9.2–13.5]	1136	12.6 [9.8–15.1]	782	12.0 [6.9–14.6]	460	11.6 [7.8–14.2]	333	7.8 [0.0–10.6]
	Male	538	10.9 [6.1–14.6]	496	16.9 [10.4–21.1]	300	13.7 [5.3–18.3]	187	13.9 [0.0–25.5]	122	18.2 [8.9–29.4]
Month of	April	168	13.5 [4.5–18.7]	162	11.3 [0.8–17.4]	94	27.3 [9.3–38.4]	53	17.4 [0.0–28.9]	31	18.5 [6.6–14.6]
start	Other months	1662	12.2 [9.5–14.0]	1471	14.0 [13.0–19.4]	992	10.9 [7.6–13.5]	581	10.8 [7.8–14.8]	424	10.5 [8.2–45.4]
Month of	April	181	13.7 [1.6–20.4]	145	13.0 [6.1–18.4]	data not available		54	13.9 [0.0–25.5]	34	14.3 [0.0–33.3]
birth	Other months	1649	10.9 [7.7–13.0]	1488	13.7 [11.5–16.3]			580	11.6 [7.8–14.2]	421	11.1 [0.0–13.6]
Assay	MPA	362	30.3 [18.6–36.1]	631	20.0 [14.7–25.1]						
method	MGA	1199	8.4 [6.9–10.6]	256	7.0 [1.3–10.7]						
	LUC			754	11.0 [8.0–14.6]						
	iLite	269	4.0 [0.7–7.4]								

Looking to all the cumulative probability curves across the countries, we observed very similar rates of ADA development by type of IFNβ-therapy.

Age at start of IFNβ-therapy was associated with immunogenicity in each country (p < 0.01) with the exception of Germany (Dusseldorf group: p = 0.45; Munich group: p = 0.08). Older patients had an increased risk of ADA. In Austria, Germany and Denmark, male patients had an immune response to IFNβ more frequently than females, this association between sex and ADA being significant in Austria and in Germany–Munich group (p < 0.01), and borderline significant for Denmark (p = 0.06). This relationship did not reach significance for Germany–Dusseldorf group (p = 0.09) and Sweden (p = 0.14). When comparing by period of treatment start, patients who started IFNβ therapy in April were at higher risk of developing ADA compared to patients who started at any other month in Germany (p < 0.0001 in Dusseldorf-group, and p = 0.07 in Munich-group). This association was not significant in Sweden (p = 0.15) and in Denmark (p = 0.48). Conversely, in Austria, patients who started IFNβ therapy in April were at lower risk of developing ADA (p = 0.04).

The month of birth was not associated with ADA development in any of the studied cohorts.

The ADA assay methods which have changed across time in Sweden and Austria were associated with ADA occurrence, suggesting a difference in the sensitivity of the assays as previously reported [18]. The MPA method detected a higher rate of ADA with 20% (95% CI 14.7–25.1) of the patients who developed ADA before 18 months of therapy in Austria and 30.3% (95% CI 18.6–36.1) in Sweden, while 7% (95% CI 1.3–10.7) developed ADA with the MGA method in Austria and 8.4% (95% CI 6.9–10.6) in Sweden. Concerning the more recent assay methods, 11% (95% CI 8.0–14.6) of the patients developed ADA before 18 months with the LUC method in Austria and 4% (95% CI 0.7–7.4) with the iLite method in Sweden.

#### Results for natalizumab

**Fig 3:**
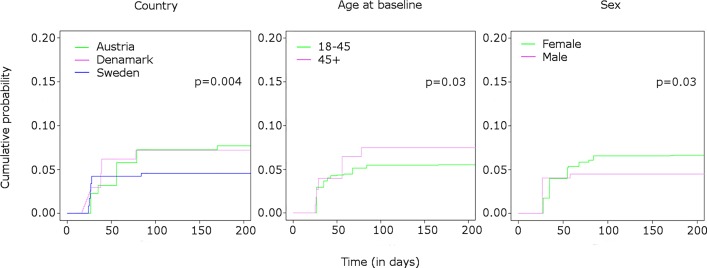
Cumulative probability of developing anti-natalizumab ADA. Estimated cumulative probability of developing anti-natalizumab ADA by categorical variables with p-values for rank-based tests adapted for interval censoring.

In the univariate analysis of the pooled data from Sweden, Denmark and Austria, 6.0% [5.1–6.8] of the patients developed anti-natalizumab ADA. As expected most of the ADA occurred within six months of natalizumab therapy. The country was associated with occurrence of ADA (p = 0.004) (Fig 3).

In Sweden 4.6% (95% CI 3.7–5.9) of the patients developed ADA before six months of therapy, while 7.2% (95% CI 5.0–8.9) and 7.7% (95% CI 5.3–9.8) did in Denmark and Austria respectively (Table 2).

**Table 2:** Estimated cumulative probabilities of ADA development before six months of natalizumab therapy.

		(N = 3440)	
		n	% 95% CI
Country	Sweden	1618	4.6 [3.7–5.9]
	Denmark	1113	7.2 [5.0–8.9]
	Austria	709	7.7 [5.3–9.8]
Age at	18–45	2585	5.5 [4.6–6.5]
baseline	45+	855	7.5 [4.9–9.5]
Sex	Female	2453	6.6 [5.6–7.8]
	Male	987	4.5 [3.2–5.9]

Age at start of natalizumab therapy was associated with ADA occurrence (p = 0.03). Among patients older than 45 years, 7.5% (95% CI 4.9–9.5) had a detectable immunogenicity at six months of therapy compared to 5.5% (95% CI 4.6–6.5) of the younger patients.

Sex was significantly associated with ADA occurrence (p = 0.03). More female patients (6.6%, 95% CI 5.6–7.8) have measurable ADA than males (4.5%, 95% CI 3.2–5.9) at six months of therapy.

None of the months of natalizumab therapy start and none of the months of birth were associated with ADA production when the countries were analyzed separately. However in the pooled analysis, patients starting the therapy in November had a higher risk of developing ADA (S3 and S4 Tables).

None of the variables characterizing previous IFNβ therapy (last IFNβ taken, first IFNβ taken, last ADA status during IFNβ therapy, total duration of IFNβ therapy and number of different IFNβ therapies) was significantly associated with development of ADA against natalizumab (S1 Fig).

### Multivariate analyses

#### Results for IFNβ

**Fig 4:**
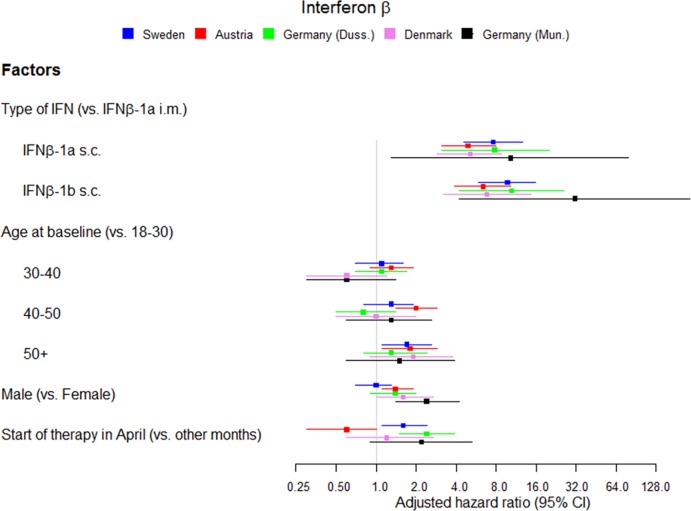
Multivariate risk factors for anti-IFNβ ADA development. Multivariate risk factors for anti-IFNβ ADA development in each cohort, adjusted on assay method at the last visit in Sweden and Austria.

Type of IFNβ, age at start of therapy, sex and month of therapy start were included in a multivariate Cox regression model for each cohort and results are reported in Table 3 and Fig 4.

**Table 3:** Adjusted hazard ratios for risk factors of anti-IFNβ ADA development.

		Sweden		Austria		Germany-(Dusseldorf)		Denmark		Germany-		Pooled*	
		(cut-off = 200 TRU/mL)		(cut-off = 100 TRU/mL)		(cut-off = 100 TRU/mL)		(cut-off = 20 TRU/mL)		(Munich)			
		(N = 1830)		(N = 1632)		(N = 1082)		(N = 618)		(N = 437)		(N = 5638)	
		HR	95% CI	HR	95% CI	HR	95% CI	HR	95% CI	HR	95% CI	HR	95% CI
Type of IFNβ	IFN-1a i.m.	1.0	reference	1.0	reference	1.0	reference	1.0	reference	1.0	reference	1.0	reference
	IFN-1a s.c.	7.6	[4.6–12.6]	4.9	[3.1–8.0]	7.8	[3.1–20.0]	5.1	[2.9–8.8]	10.3	[1.3–79.3]	6.4	[4.9–8.4]
	IFN-1b s.c.	9.7	[5.9–15.8]	6.4	[3.9–10.2]	10.4	[4.2–25.9]	6.8	[3.2–14.5]	31.2	[4.2–229.5]	8.7	[6.6–11.4]
Age at baseline	18–30	1.0	reference	1.0	reference	1.0	reference	1.0	reference	1.0	reference	1.0	reference
	30–40	1.1	[0.7–1.6]	1.3	[0.9–1.9]	1.1	[0.7–1.7]	0.6	[0.3–1.2]	0.6	[0.3–1.4]	1.1	[0.8–1.3]
	40–50	1.3	[0.8–1.9]	2.0	[1.4–2.9]	0.8	[0.5–1.4]	1.0	[0.5–2.0]	1.3	[0.6–2.6]	1.3	[1.1–1.6]
	50+	1.7	[1.1–2.6]	1.8	[1.1–2.9]	1.3	[0.8–2.4]	1.9	[0.9–3.8]	1.5	[0.6–3.9]	1.8	[1.4–2.3]
Sex	Female	1.0	reference	1.0	reference	1.0	reference	1.0	reference	1.0	reference	1.0	reference
	Male	1.0	[0.7–1.3]	1.4	[1.1–1.9]	1.4	[0.9–2.0]	1.6	[1.0–2.7]	2.4	[1.4–4.2]	1.3	[1.1–1.6]
Month of start	Other months	1.0	reference	1.0	reference	1.0	reference	1.0	reference	1.0	reference	1.0	reference
	April	1.6	[1.1–2.4]	0.6	[0.3–1.0]	2.4	[1.5–3.9]	1.2	[0.6–2.7]	2.2	[0.9–5.3]	1.3	[1.0–1.6]
Assay	MPA	1.0	reference	1.0	reference								
method	MGA	0.3	[0.2–0.5]	0.5	[0.3–0.9]								
	LUC			0.8	[0.6–1.0]								
	iLite	0.2	[0.1–0.3]										

*Analysis adjusted on the country

As the ADA assay method was acting as a confounding factor for the Swedish and Austrian cohorts, we adjusted for this variable in the Cox model. The adjusted hazard ratios (HR) varied from 4.9 to 7.8 for IFNβ-1a s.c. compared to IFNβ-1a i.m. (95% CI ranging from 2.9 to 20.0) and from 6.4 to 10.4 for IFNβ-1b s.c. compared to IFNβ-1a i.m. (95% CI ranging from 3.2 to 25.9) (Table 3 and Fig 4).

HRs relative to age older than 50 compared to age between 18 and 30 varied from 1.3 to 1.9 across the cohorts (95% CI ranging from 0.8 to 3.8) (Table 3 and Fig 4).

The adjusted HRs for males compared to females were significant in Austria (1.4, 95% CI 1.1–1.9) and Denmark (1.6, 95% CI 1.0–2.7), at the limit of significance in Germany-Dusseldorf (1.4, 95% CI 0.9–2.0) and not significant in Sweden (1.0, 95% CI 0.7–1.3) (Table 3 and Fig 4).

After adjustment for other risk factors, start of therapy in April compared to start in any other month was still a risk factor for ADA development in Sweden (1.6, 95% CI 1.1–2.4) and Germany (Dusseldorf) (2.4, 95% CI 1.5–3.9), and was associated with a lower risk in Austria (0.6, 95% CI 0.3–1.0) (Table 3 and Fig 4).

According to the analysis of the pooled dataset of the five cohorts we found similar associations with type of treatment (HR = 6.4 [4.9–8.4] for IFNβ-1a s.c. and 8.7 [6.6–11.4] for IFNβ-1b s.c. both versus IFNβ-1a i.m.), age (HR = 1.8 [1.4–2.3] for patients older than 50 versus 18–30), sex (HR = 1.3 [1.1–1.6] for males versus females), month of therapy start (HR = 1.3 [1.0–1.6] for April compared to other months) (Table 3).

#### Results for natalizumab

**Fig 5:**
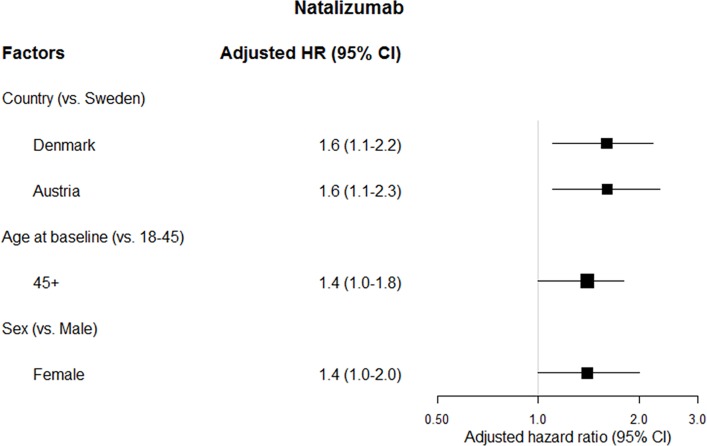
Multivariate risk factors for anti-natalizumab ADA development. (N = 3440).

Age at start of natalizumab therapy and sex were analyzed together with a Cox regression model for each cohort and for the pooled dataset with adjustment on the country (Table 4 and Fig 5). The two variables were identified as risk factors for ADA occurrence in the pooled cohort, whereas the associations with ADA development were not statistically significant in each individual cohort.

**Table 4:** Adjusted hazard ratios for risk factors of anti-natalizumab ADA development.

		Sweden		Denmark		Austria		Pooled*	
		(N = 1618)		(N = 1113)		(N = 709)		(N = 3440)	
		HR	95% CI	HR	95% CI	HR	95% CI	HR	95% CI
Age at	18–45	1.0	reference	1.0	reference	1.0	reference	1.0	reference
baseline	> = 45	1.2	[0.7–2.0]	1.4	[0.9–2.2]	1.6	[0.8–3.0]	1.4	[1.0–1.8]
Sex	Female	1.0	reference	1.0	reference	1.0	reference	1.0	reference
	Male	0.7	[0.4–1.3]	0.8	[0.5–1.3]	0.6	[0.3–1.2]	0.7	[0.5–0.98]

*Analysis adjusted on the country

HRs for age older than 45 at start of therapy compared to age 18–45 varied from 1.2 to 1.6 across the cohorts (95% CI ranging from 0.7 to 3.0) and was 1.4 (95% CI 1.0–1.8) over all the cohorts pooled together.

The HR for men compared to women was 0.7 (95% CI 0.4–1.3) in Sweden, 0.8 (95% CI 0.5–1.3) in Denmark and 0.6 (95% CI 0.3–1.2) in Austria. In the pooled dataset, it was 0.7 (95% CI 0.5–0.98) (Table 4 and Fig 5).

## Discussion

To the best of our knowledge, this is the first collaborative cohort analysis investigating the impact of patient characteristics and type of BPs on the occurrence of ADA in MS. In order to carry out an unbiased evaluation of the relationships between bio-clinical factors and the occurrence of ADAs, we have selected a target population which was extracted from the whole ABIRISK database. This selected population is composed by naive adult patients with biological samples being taken within the first two years of treatment. From this selected population which combines data from four national European reference laboratories and one research center, we report that the occurrence of ADA against BPs used to treat MS (IFNβ and natalizumab) were associated with age and sex. Older patients were at higher risk of developing ADA against both IFNβ and natalizumab. In contrast, the sex effect differs between the two BPs, with male patients being more at risk of anti-IFNβ ADA and female patients more at risk of anti-natalizumab ADA.

We confirm the previously reported differences in the cumulative probability of ADA occurrence at 18 months of treatment for the different types of IFNβ (ranging between 1.1–4.2% for IFNβ-1a i.m., 11.1–23.0% for IFNβ-1a s.c. and 17.4–33.3% for IFNβ-1b s.c. in the present study) [1].

Interestingly, we also observe that patients who started IFNβ therapy in the month of April showed a higher risk of ADA development. This relationship is not seen in every cohort but stays significant in the German (Dusseldorf) and in the Swedish cohorts after adjustment for multiple testing. We do not find a relationship between ADA occurrence and the month of birth neither for IFNβ nor for natalizumab-treated patients. In the latter, no factor related to previous IFNβ exposure (last IFNβ taken, first IFNβ taken, last ADA status during IFNβ therapy, total duration of IFNβ therapy and number of different IFNβ therapies) is associated to anti-natalizumab ADA development.

Since this study relies on historical cohorts, we had to cope with various follow-up schemes. For example, in Sweden and Denmark the assays were done routinely, while in Germany the testing was left to the discretion of the treating physician. However, from a previous study, the characteristics of the patients who have been tested due to an indication of worsening disease and therefore a suspicion of ADA did not differ significantly in ADA frequency from those that were tested according to regular routine dates [27]. Moreover, in our analyses we observed similar follow-up schemes by age and sex.

In this study, we do not know the exact time of ADA occurrence but we know whether or not ADA has occurred within a monitoring interval. These so-called interval censored data have often been analyzed with various ad-hoc solutions such as the use of either the right-sided endpoint for subject with positive ADA or the midpoint in the interval. These solutions are known to be non-optimal and biased when the times between visits are long or the frequency of ADA assessment varies in the population which is the case in our study that included various cohorts with different follow-up scheme. Here, all the analyses were performed using methodologies designed for such interval-censored data.

In this study, it is worth noting that different types of assay were sequentially used over time and that these differences might have reflected changes in treatment choice over time or changes in treatment formulation as for the IFNβ-1a s.c. in 2007 [17]. We cannot assess the effect of change in IFNβ-1a s.c. formulation as its date is too close to the date of change in type of assay used. Nevertheless when we adjusted on the type of IFNβ treatment in the multivariate analyses, differences between the assay methods in terms of ADA development remained significant, suggesting that assay methods may differ in sensitivity of ADA detection as previously reported [18].

Due to the wide range of ADA detection process for IFNβ, the analyses were initially performed separately on each country. Access to reference laboratory data enabled us to have sufficient number of patients in each country that gave us sufficient power to detect the association of interest if it existed. Since we did not observe discrepancies between the results obtained for each country, we then also performed the multivariate analysis on the pooled cohort.

For natalizumab, since the same assay method was used and the laboratories had been tested yearly for concordant test results of 20 samples (personal communication from A. Fogdell-Hahn), the univariate analysis was applied on the pooled cohort. As we observed a country effect, multivariate analysis was performed both on separate and pooled cohort.

Our selection criteria implied that we included more patients who began their treatment more recently. This led for instance to the fact that our study population includes more IFNβ-1a i.m. treated patients than the global ABIRISK database. Because of the differences between included and excluded patients in terms of type of treatment, age at first treatment or first ADA status, our study population might not be representative of the general population of the IFNβ-treated or natalizumab-treated and ADA tested MS patients. This necessary selection in order to cope with the two-year ADA occurrence window could influence the global prevalence of ADA development, but not the tests of comparisons between groups and the measure of associations between potential risk factors and ADA development. Another consequence of the selection is that we might lose power in some less represented subcategories. For instance, older patients in Germany (Dusseldorf) were less included because it might have been more difficult to track their date of first dose, which may explain why the association with age could not be seen.

The differences among treatments that we observed were in the same range and in the same direction as those described in previous studies [1]. The higher immunogenicity of IFNβ-1b compared to IFNβ-1a has been attributed to its lack of glycosylation which makes it highly prone to form aggregates. This has been confirmed in an animal model, where aggregated IFNβ-1b and IFNβ-1a induced higher ADA seroprevalence than non-aggregated IFNβ-1a [28]. Animal studies have also suggested that both a T-independent and a T-dependent response might contribute to IFNβ-1b immunogenicity, where the T-independent response is characterized by low ADA titers and low ADA persistency due to lack of a memory response [29]. Conversely, IFNβ-1a immunogenicity, although less frequent, generates higher titer and persistent ADA responses, often with a neutralizing activity [15,21]. In our study as in previous ones, although risk of ADA occurrence was higher in IFNβ-1b s.c. treated MS-patients than in IFNβ-1a i.m. treated, the difference with IFNβ-1a s.c.-treated was not significant.

We found a significantly higher immunogenicity of both IFNβ and natalizumab in older patients. The impact of age was assessed in previous studies but was not found to be associated with ADA development [30,31]. However a higher median age was observed in ADA positive compared to ADA negative patients in one study (45 years versus 43 years, p<0.0001, n = 1115) [15]. The negative studies had lower number of patients (n = 301 and n = 541) and may have lacked in power to detect the association.

As thymic function declines after puberty, the main source of naïve T cells to counterbalance T-cell turnover in adult life comes from their homeostatic proliferation [32]. Mathematical models corroborated by experimental data postulate that in spite of T-cell turnover, homeostatic mechanisms keep a constant T-cell pool and the maintenance of T-cell repertoire diversity is IL7- and TCR-dependent [33–35]. Continuous loss of senescent T-cells and conversion of naïve T cells into memory T cells due to pathogen exposure progressively induce a reduction of the naïve T-cell repertoire diversity and lymphopenia with a consequent decrease in adaptive immune responses against novel antigens and increase in homeostatic proliferation in the elderly [36]. The lymphopenic condition is known to induce a stronger proliferation of high affinity autoreactive effector T cells to self-antigens, and if not compensated by an appropriate proliferation or novel thymic production of regulatory T cells it may easily lead to development of autoimmune responses [37]. Interestingly, more than 50% of alemtuzumab-treated MS-patients experience secondary autoimmune manifestations as a consequence of lymphopenia, and development of autoimmunity is strongly associated with insufficient recovery of thymopoiesis after a strong initial homeostatic proliferation [38]. Similarly, lymphopenia induced autoimmunity might explain the increased risk of anti-IFNβ ADA development in older MS-patients: chronic stimuli with IFNβ might induce homeostatic proliferation of effector T cells against a self-antigen not paralleled by an appropriate thymopoiesis of novel regulatory T cells.

In this study we identify a significant association of sex with ADA occurrence, with men being more susceptible to develop IFNβ ADA. In one study about immunogenicity of IFNβ in MS, it was observed that a higher proportion of female than male patients became ADA positive after 12 months of treatment with IFNβ-1a subcutaneously [39]. Later this result was not confirmed as sex was not a predictor of development of neutralizing antibodies in clinical data collected prospectively in Denmark [31]. Apart from these studies, the association between sex-related factors and ADA development has not been investigated so far. Their characterization is of utmost importance since some of these factors may be controlled (e.g. oral contraception, breastfeeding) for prevention of ADA development. A thorough knowledge of X-linked genetic factors, hormonal factors and behavioral factors is needed to distinguish which sex- (or gender-) specific factor mostly contributes to this difference. Among interesting gene candidates encoded on the X-chromosome are several transcription factors such as FOXP3 and immunity receptors such as TLR7 and TLR8. Sex hormones are important modulators of immune responses and several autoimmune diseases display a sex bias with a higher prevalence in women [40]. The hormonal status of the patient at therapy start is therefore likely to influence the development of ADA. Interestingly the sex difference in IFNβ ADA occurrence was observed in all the analyzed European countries except Sweden. A hypothesis to account for this peculiarity might be a local behavioral difference influencing a relevant exposure such that the exposure would be similar in men and women in Sweden, but higher in men in the other countries.

Concerning natalizumab, inversely to IFNβ, we observed that women developed ADA more often than men. IFNβ being a cytokine and natalizumab a monoclonal antibody, they are structurally different and might have distinct immunogenicity mechanisms. A slower pharmacokinetics in women than in men has been reported for several drugs, including the TNFα-blocking monoclonal antibody adalimumab [41], and might induce a prolonged exposure to the drug in women with possible effects on immunogenicity. The early pharmacokinetics study on natalizumab did not analyze the influence of sex [42], and a sex difference in natalizumab clearance warrants further investigation.

An association of month of birth with risk of developing MS has clearly been established in several case-control studies from the Northern and Southern hemisphere, albeit with a small effect size [43]. In the Northern hemisphere individuals born in April-May carry a higher risk of MS, whereas individuals born in October-November display a slight protection from MS. The month of birth effect is thought to derive from fetal exposure to a seasonal factor in the last trimester of pregnancy, a crucial period in the negative selection of autoreactive T cells in the fetal thymus [44]. As ADA (in particular against IFNβ) may be considered an autoimmune phenomenon, we investigated whether a month of birth effect could be linked also to ADA occurrence, but we were not able to detect an association. Considering the small effect size which has been reported for the association between month of birth and MS (OR = 1.08 for May in a UK case-control study [45]), we did not have the power to detect a similarly small effect in our study.

Instead we observed a significant association of the month of therapy start with IFNβ ADA in Sweden, Germany (Dusseldorf group), Austria, and borderline significant in Germany (Munich group). For all the cohorts the month associated with ADA was April, but surprisingly start in April conferred an increased risk in all the cohorts except Austria, where it conferred decreased risk. Further investigations are warranted to confirm this association and to identify the underlying seasonal factor. Candidate seasonal factors which might influence the immune status at start of therapy are allergy causing spring pollens or early spring vitamin D insufficiency.

Vitamin D is a modulator of T- and B-cell responses [46], and genetically and environmentally determined vitamin D insufficiency is a risk factor for MS [47,48]. B-lymphocytes express the VDR and vitamin D is able to suppress their proliferation, immunoglobulin production, class switch and differentiation to plasma cells [49], thus it might play a role in ADA development. It has been suggested that part of the effect of IFNβ is mediated by induction of vitamin D synthesis [50]. From these results and from the previous knowledge of the immunomodulatory activity of vitamin D it could be hypothesized that vitamin D levels at start of therapy, influenced by the seasonal UV-radiation and possibly also by early response to IFNβ therapy, is a factor associated with ADA development. Several randomized controlled clinical trials on vitamin D supplementation to IFNβ or glatiramer therapy in MS are ongoing [51,52]. Studies to correlate vitamin D levels before and during IFNβ therapy with later ADA development are warranted.

In conclusion, the findings obtained from this consortium cohort provide new insights on ADA occurrence. Age and sex were found to be risk factors of ADA development against IFNβ and natalizumab MS therapies in data from four national European reference laboratories and one research study. We also observed an association with the month of start of therapy which warrants further investigation. The next step of our research project, which is currently ongoing, is to include data from additional MS registries and research study questionnaires to assess other potential explanatory factors such as vitamin D serum levels, previous and concomitant medications, medical history, infections, vaccinations, tobacco consumption and genetic factors. The effect size of the associations with age and sex were not high enough to discourage clinicians in terms of drug prescription, but a more frequent ADA monitoring is recommended for the risk groups to allow earlier detection of ADA and an appropriate drug switch.

## Supporting Information

S1 Fig Association of variables characterizing previous IFNβ therapy with anti-natalizumab ADA occurrence risk. (TIF)

S1 File List of ABIRISK Consortium partners and members. (DOCX)

S1 Table Anti-IFNβ ADA development hazard ratios with 95% confidence intervals for each month of start of therapy (versus all the other months). (DOCX)

S2 Table Anti-IFNβ ADA development hazard ratios with 95% confidence intervals for each month of birth (versus all the other months). (DOCX)

S3 Table Anti-natalizumab ADA development hazard ratios with 95% confidence intervals for each month of start of therapy (versus all the other months). (DOCX)

S4 Table Anti-natalizumab ADA development hazard ratios with 95% confidence intervals for each month of birth (versus all the other months). (DOCX)

S5 Table Comparison of the of the included to the excluded IFNβ-treated patients. (DOCX)
