# Supplementary figures and images for: Occurrence of Anti-Drug Antibodies against Interferon-Beta and Natalizumab in Multiple Sclerosis: A Collaborative Cohort Analysis
Source: PLoS One. 2016 Nov 2;11(11):e0162752. doi: 10.1371/journal.pone.0162752 (PMC5091903; doi:10.1371/journal.pone.0162752)

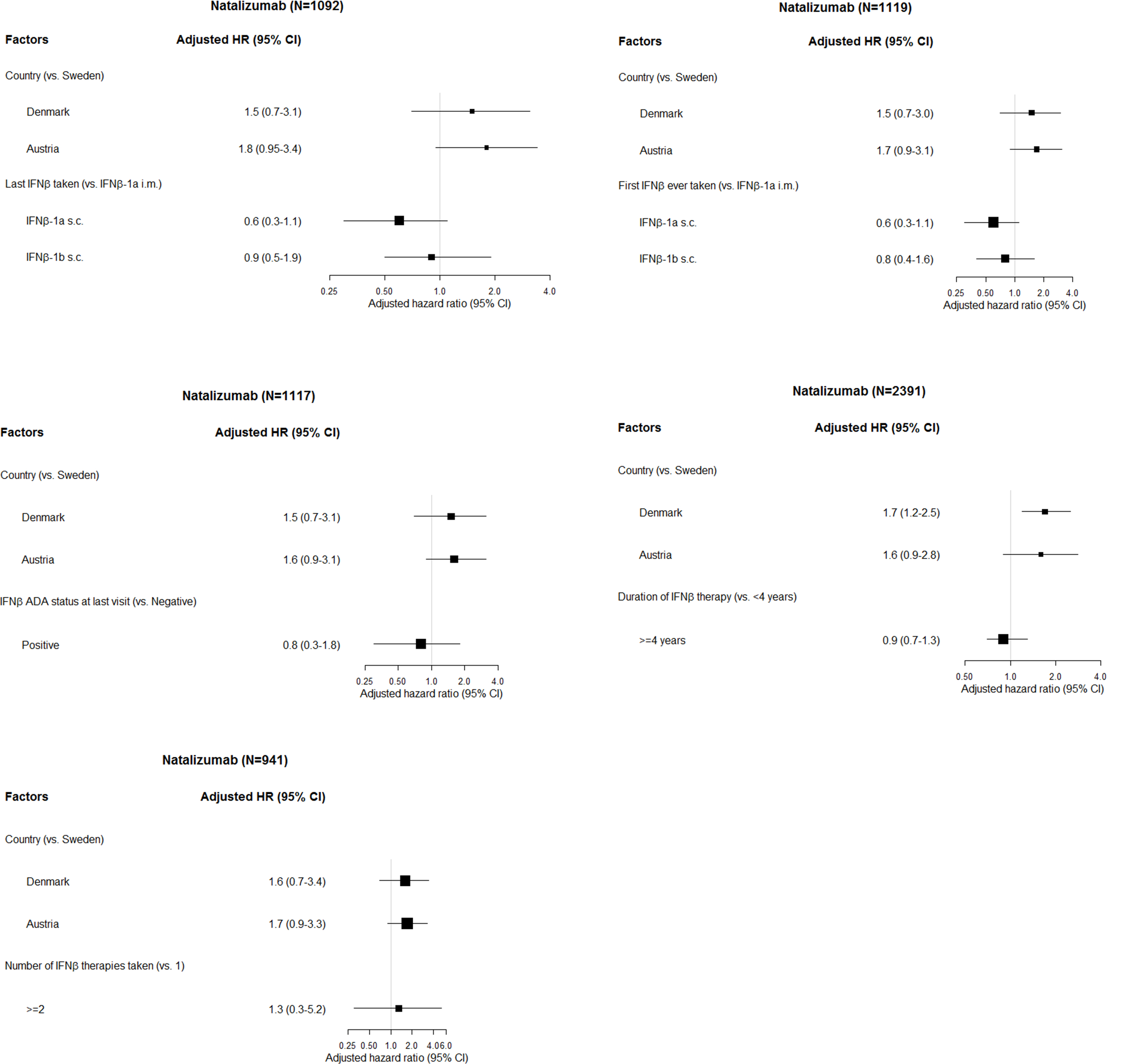

Supplement: S1 Fig — (TIF) [file pone.0162752.s001.tif]
