# Supplementary material for: Occurrence of Anti-Drug Antibodies against Interferon-Beta and Natalizumab in Multiple Sclerosis: A Collaborative Cohort Analysis
Source: PLoS One. 2016 Nov 2;11(11):e0162752. doi: 10.1371/journal.pone.0162752 (PMC5091903; doi:10.1371/journal.pone.0162752)
Supplement: S1 File — (DOCX) [file pone.0162752.s002.docx]

**List of ABIRISK partners, leaders and task responsible persons**

Medizinische Universität Innsbruck (Florian Deisenhammer), UCB Pharma SA (Louis Christodoulou), GlaxoSmithKline (Dan Sikkema, Amy Loercher, Julie Davidson, Andy Lawton, Steve Etheridge, Sally Miles), INSERM (Marc Pallardy, Sophie Tourdot, Xavier Mariette, Sebastien Lacroix-Desmazes, Philippe Broet , Delphine Bachelet, Nadia El-Hamdi), Academisch Medisch Centrul bij de Universiteit van Amsterdam (Niek De Vries, Anne Musters), Assistance Publique Hopitaux de Paris (Aline Doublet), Groupe d’études thérapeutiques des affections inflammatoires du tube digestif (Matthieu Allez, Sabrina Williams), Universitaetsklinikum Bonn (Johannes Oldenburg, Thilo Albert), Karolinska Institutet (Anna Fogdell Hahn, Malin Ryner, Ryan Ramanujam), Pfizer (Tim Hickling), Merck Serono (Elisa Bertotti), Ipsen (Julie Le Grand), University College London (Claudia Mauri, Liz Jury), Sanofi-Aventis Research and Development (Vincent Mikol, Agnès Hincelin-Mery, Catherine Prades, Pauline Loas), Università di Firenze (Enrico Maggi), Novartis Pharma AG (Annette Karle, Sebastian Spindeldreher, Verena Romach-Riegraf), Fondazione per l’Istituto di Ricerca in Biomedicina (Antonio Lanzavecchia), Klinikum rechts der Isar der Technischen Universitaet Muenchen (Bernhard Hemmer), Commissariat à l’Energie Atomique (Bernard Maillere), Novo Nordisk (Christian Ross Pedersen), Scicross AB (Pierre Dönnes), Bayer Schering Pharma AG (Jeannette Lo, Pascale Buchmann), eTRIKS (Fabien Richard), Paul-Ehrlich-Institut (Christine Keipert), ALTA Ricerca e Sviluppo in Biotecnologie S.r.l.u. (Riccardo Bertini, Simona Farnetani).
