# Supplementary material for: Occurrence of Anti-Drug Antibodies against Interferon-Beta and Natalizumab in Multiple Sclerosis: A Collaborative Cohort Analysis
Source: PLoS One. 2016 Nov 2;11(11):e0162752. doi: 10.1371/journal.pone.0162752 (PMC5091903; doi:10.1371/journal.pone.0162752)
Supplement: S1 Table — (DOCX) [file pone.0162752.s003.docx]

**S1 Table**

|  | Sweden  (cut-off=200 TRU/ml)  (N=1830) | | Austria (cut-off=100 TRU/mL)  (N=1633) | | Germany (Dusseldorf)  (cut-off=100 TRU/mL)  (N=1086) | | Denmark  (cut-off=20 TRU/mL)  (N=634) | | Germany (Munich)  (N=455) | |
| --- | --- | --- | --- | --- | --- | --- | --- | --- | --- | --- |
|  | HR | 95% CI | HR | 95% CI | HR | 95% CI | HR | 95% CI | HR | 95% CI |
| January | 1.1 | [0.7-1.7] | 1.0 | [0.5-2.4] | 1.4 | [0.8-2.5] | 0.3 | [0.1-1.2] | 0.9 | [0.3-2.9] |
| February | 1.3 | [0.8-2.1] | 0.9 | [0.6-1.5] | 1.2 | [0.6-2.1] | 1.3 | [0.6-2.9] | 0.8 | [0.3-2.7] |
| March | 0.6 | [0.3-1.1] | 0.9 | [0.6-1.4] | 1.0 | [0.5-2.0] | 1.7 | [0.9-3.2] | 1.6 | [0.6-3.9] |
| April | 1.5 | [1.0-2.2] | 0.6 | [0.3-1.0] | 2.4 | [1.5-3.9] | 1.3 | [0.6-2.9] | 2.0 | [0.9-4.5] |
| May | 0.6 | [0.3-1.0] | 1.3 | [0.8-1.9] | 0.6 | [0.3-1.2] | 0.4 | [0.1-1.5] | 1.0 | [0.4-2.5] |
| June | 0.9 | [0.6-1.5] | 1.2 | [0.7-1.8] | 0.6 | [0.3-1.4] | 1.4 | [0.7-2.8] | 1.9 | [0.8-4.2] |
| July | 1.1 | [0.6-2.1] | 0.6 | [0.3-1.1] | 1.4 | [0.7-2.6] | 1.2 | [0.5-2.9] | 0.6 | [0.2-2.0] |
| August | 0.8 | [0.5-1.5] | 1.2 | [0.8-1.9] | 1.0 | [0.5-1.8] | 1.3 | [0.5-2.9] | 1.0 | [0.4-2.6] |
| September | 1.2 | [0.7-1.8] | 1.2 | [0.8-2.0] | 0.9 | [0.5-1.8] | 0.3 | [0.1-1.3] | 0.4 | [0.1-1.5] |
| October | 1.0 | [0.6-1.5] | 1.0 | [0.6-1.6] | 0.8 | [0.4-1.6] | 1.0 | [0.4-2.3] | 0.4 | [0.09-1.5] |
| November | 1.2 | [0.7-1.8] | 0.8 | [0.5-1.4] | 0.5 | [0.2-1.3] | 1.3 | [0.6-2.8] | 0.7 | [0.2-2.1] |
| December | 0.8 | [0.5-1.4] | 1.5 | 1.0-2.4] | 0.6 | [0.2-1.4] | 0.6 | [0.2-2.0] | 1.6 | [0.7-3.7] |
