# Supplementary material for: Occurrence of Anti-Drug Antibodies against Interferon-Beta and Natalizumab in Multiple Sclerosis: A Collaborative Cohort Analysis
Source: PLoS One. 2016 Nov 2;11(11):e0162752. doi: 10.1371/journal.pone.0162752 (PMC5091903; doi:10.1371/journal.pone.0162752)
Supplement: S4 Table — (DOCX) [file pone.0162752.s006.docx]

**S4 Table**

|  | Pooled (N=3440) | |
| --- | --- | --- |
|  | HR | 95% CI |
| January | 1.3 | [0.8-2.0] |
| February | 0.9 | [0.5-1.6] |
| March | 1.2 | [0.7-1.9] |
| April | 0.7 | [0.4-1.3] |
| May | 1.1 | [0.7-1.8] |
| June | 1.2 | [0.8-1.9] |
| July | 1.0 | [0.6-1.7] |
| August | 0.8 | [0.5-1.4] |
| September | 1.0 | [0.6-1.7] |
| October | 1.1 | [0.7-1.7] |
| November | 0.9 | [0.6-1.6] |
| December | 0.8 | [0.5-1.3] |
