# Supplementary material for: Occurrence of Anti-Drug Antibodies against Interferon-Beta and Natalizumab in Multiple Sclerosis: A Collaborative Cohort Analysis
Source: PLoS One. 2016 Nov 2;11(11):e0162752. doi: 10.1371/journal.pone.0162752 (PMC5091903; doi:10.1371/journal.pone.0162752)
Supplement: S5 Table — (DOCX) [file pone.0162752.s007.docx]

**S5 Table**

|  |  | Austria | | | | | Denmark | | | | | Sweden | | | | | | Germany (Dusseldorf) | | | | | | Germany (Munich) | | | | |
| --- | --- | --- | --- | --- | --- | --- | --- | --- | --- | --- | --- | --- | --- | --- | --- | --- | --- | --- | --- | --- | --- | --- | --- | --- | --- | --- | --- | --- |
|  |  | included  (N=1633) | | excluded  (N=2248) | |  | included  (N=634) | | excluded  (N=340) | |  | included  (N=1823) | | excluded  (N=3187) | |  | included  (N=1086) | | | excluded  (N=4399) | |  | included  (N=455) | | | excluded  (N=1800) | |  |
|  |  | n | % | n | % | p* | n | % | n | % | p* | n | % | n | % | p* | n | | % | n | % | p* | n | | % | n | % | p* |
| Age at baseline | 18-30 | 491 | 30 | 261 | 25 | 0.03 | 147 | 24 | 97 | 30 | 0.14 | 387 | 21 | 312 | 15 | <0.0001 | 373 | | 34 | 903 | 28 | 0.0004 | 129 | | 28 | 392 | 25 | 0.013 |
|  | 30-40 | 574 | 35 | 368 | 36 |  | 212 | 34 | 109 | 34 |  | 560 | 31 | 710 | 34 |  | 339 | | 31 | 1082 | 34 |  | 150 | | 33 | 611 | 39 |  |
|  | 40-50 | 416 | 25 | 294 | 28 |  | 177 | 29 | 76 | 24 |  | 550 | 30 | 669 | 32 |  | 263 | | 24 | 901 | 28 |  | 146 | | 32 | 434 | 27 |  |
|  | 50+ | 152 | 9 | 113 | 11 |  | 82 | 13 | 39 | 12 |  | 326 | 18 | 429 | 21 |  | 111 | | 10 | 286 | 9 |  | 30 | | 7 | 149 | 9 |  |
|  | <18 | 0 |  | 53 |  |  | 0 |  | 21 |  |  | 0 |  | 33 |  |  | 0 | |  | 76 |  |  | 0 | |  | 38 |  |  |
|  | NA | 0 |  | 1159 |  |  | 16 |  | 19 |  |  | 0 |  | 1067 |  |  | 0 | |  | 1151 |  |  | 0 | |  | 176 |  |  |
|  |  |  |  |  |  |  |  |  |  |  |  |  |  |  |  |  |  | |  |  |  |  |  | |  |  |  |  |
| Sex | Female | 1136 | 70 | 1514 | 69 | 0.60 | 450 | 71 | 247 | 73 | 0.63 | 1290 | 71 | 2290 | 72 | 0.41 | 782 | | 72 | 2914 | 71 | 0.54 | 333 | | 73 | 1283 | 71 | 0.46 |
|  | Male | 496 | 30 | 688 | 31 |  | 184 | 29 | 93 | 27 |  | 533 | 29 | 897 | 28 |  | 300 | | 28 | 1175 | 29 |  | 122 | | 27 | 513 | 29 |  |
|  | NA | 1 |  | 46 |  |  | 0 |  | 0 |  |  | 0 |  | 0 |  |  | 4 | |  | 310 |  |  | 0 | |  | 4 |  |  |
|  |  |  |  |  |  |  |  |  |  |  |  |  |  |  |  |  |  | |  |  |  |  |  | |  |  |  |  |
| First IFNβ | IFNbeta-1a i.m. | 607 | 37 | 657 | 29 | <0.0001 | 438 | 69 | 176 | 52 | <0.0001 | 873 | 48 | 965 | 34 | <0.0001 | 308 | | 28 | 1155 | 28 | 0.0003 | 130 | | 29 | 411 | 24 | 0.11 |
|  | IFNbeta-1a s.c. | 434 | 27 | 721 | 32 |  | 165 | 26 | 102 | 30 |  | 461 | 25 | 1126 | 39 |  | 380 | | 35 | 1339 | 33 |  | 154 | | 34 | 645 | 38 |  |
|  | IFNbeta-1b s.c. | 592 | 36 | 870 | 39 |  | 31 | 5 | 62 | 18 |  | 489 | 27 | 783 | 27 |  | 398 | | 37 | 1607 | 39 |  | 171 | | 37 | 625 | 37 |  |
|  | Other | 0 |  | 0 |  |  | 0 |  | 0 |  |  | 0 |  | 55 |  |  | 0 | |  | 29 |  |  | 0 | |  | 26 |  |  |
|  | NA | 0 |  | 0 |  |  | 0 |  | 0 |  |  | 0 |  | 258 |  |  | 0 | |  | 269 |  |  | 0 | |  | 93 |  |  |
| Start of therapy in April | April | 162 | 10 | 104 | 9 | 0.76 | 53 | 8 | 28 | 9 | 1 | 168 | 9 | 179 | 9 | 0.52 | 94 | | 9 | 113 | 4 | 0.91 | 31 | | 37 | 143 | 10 | 0.03 |
|  | Rest of the months | 1471 | 90 | 992 | 91 |  | 581 | 92 | 298 | 91 |  | 1655 | 91 | 1896 | 91 |  | 992 | | 91 | 1226 | 39 |  | 424 | | 37 | 1261 | 90 |  |
|  | NA | 0 |  | 1152 |  |  | 0 |  | 14 |  |  | 0 |  | 1112 |  |  | 0 | |  | 3060 |  |  | 0 | |  | 397 |  |  |
| First ADA status | Negative | 1326 | 85 | 1787 | 82 | 0.009 | 593 | 94 | 312 | 92 | 0.37 | 1666 | 92 | 2783 | 88 | 0.0001 | 784 | | 72 | 3469 | 79 | <0.0001 | 404 | | 89 | 1627 | 90 | 0.31 |
|  | Positive | 229 | 15 | 392 | 18 |  | 41 | 6 | 28 | 8 |  | 154 | 8 | 378 | 12 |  | 302 | | 28 | 930 | 21 |  | 51 | | 11 | 73 | 4 |  |
|  | NA | 78 |  | 69 |  |  | 0 |  | 0 |  |  | 3 |  | 26 |  |  | 0 | |  | 0 |  |  | 0 | |  | 0 |  |  |

*Chi-square test
